# The Canadian Nosocomial Infection Surveillance Program: Keeping an eye on antimicrobial resistance in Canadian hospitals since 1995

**DOI:** 10.14745/ccdr.v48i1112a03

**Published:** 2022-11-03

**Affiliations:** 1 Centre for Communicable Diseases and Infection Control, Public Health Agency of Canada, Ottawa, ON

**Keywords:** antimicrobial resistance, Canada, hospitals, surveillance, healthcare-associated infections, community-associated infections, antimicrobial resistant organisms, Canadian Nosocomial Infection Surveillance Program

## Abstract

Surveillance is essential to inform evidence-based policy and control measures that combat antimicrobial resistance (AMR). The Canadian Nosocomial Infection Surveillance Program (CNISP) collaborates with 88 sentinel hospitals across Canada to conduct prospective surveillance of infections and antimicrobial resistant organisms important to hospital infection prevention and control. This article aims to increase awareness of CNISP hospital-based surveillance activities. Since its inception in 1995, the scope of CNISP has expanded to include community-associated infections, outpatient *Clostridioides difficile* infections, viral respiratory infections such as coronavirus disease 2019, and emerging pathogens such as *Candida auris*. This change in scope, along with expansion to include rural, northern and community hospitals, has improved the generalizability of CNISP surveillance data. To generate actionable surveillance data, CNISP integrates demographic and clinical data abstracted from patient charts with molecular and microbiological data abstracted from laboratory testing. These data serve as a benchmark for participating hospitals and stakeholders to assess the burden of AMR in hospital and intervene as needed. Further, CNISP surveillance data are now available on a public-facing data blog that provides interactive visualizations and data syntheses sooner than peer-reviewed publications. Future directions of CNISP include the Simplified Dataset, which will capture aggregate AMR data from hospitals outside of the CNISP network, surveillance in long-term care facilities and a fourth point prevalence survey. Given its strengths and future directions, CNISP is well positioned to serve as the reference point for hospital-based AMR data in Canada.

## Introduction

Antimicrobial resistance (AMR) is a threat to global public health. Surveillance is an essential pillar of the World Health Organization global action plan to combat AMR and a key component of the Pan-Canadian Framework for Action, which provides the context and foundation to guide a pan-Canadian response to combat AMR (1,2). Both community and hospital-based surveillance are needed to inform evidence-based action, such as antimicrobial stewardship (3). We provide an overview of the Canadian Nosocomial Infection Surveillance Program (CNISP)—a hospital-based surveillance system. In describing its scope, functions and future directions, we aim to increase awareness of the CNISP hospital-based surveillance activities that contribute to combatting AMR in Canada.

### Structure

Prompted by a World Health Organization recommendation focused on combatting AMR, Health Canada established and fully funded CNISP as a hospital-based surveillance system in 1995. CNISP is a collaboration between the Public Health Agency of Canada, including the National Microbiology Laboratory, the Association of Medical Microbiology and Infectious Disease Canada and sentinel hospitals across Canada.

### Scope

**Figure 1:**
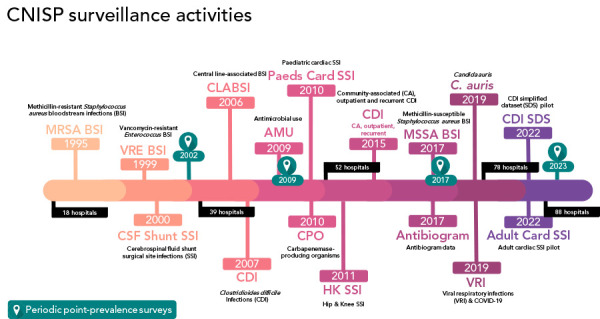
Summary of the Canadian Nosocomial Infection Surveillance Program surveillance activities, 1995 to 2022 Abbreviations: Adult Card, Adult cardiac; AMU, antimicrobial use; BSI; bloodstream infection; CA, community-associated; *C. auris*, *Candida auris*; CDI, *Clostridioides difficile* infection; CLABSI, central line-associated bloodstream infection; CPO, carbapenemase-producing organism; CSF, cerebrospinal fluid shunt; HK, hip and knee; MRSA, methicillin-resistant *Staphylococcus aureus*; MMSA, methicillin-susceptible *Staphylococcus aureus*; Paeds Card, paediatric cardiac; SDS, simplified dataset; SSI, surgical site infection; VRE, vancomycin-resistant *Enterococci*; VRI, viral respiratory infection

**Figure 2:**
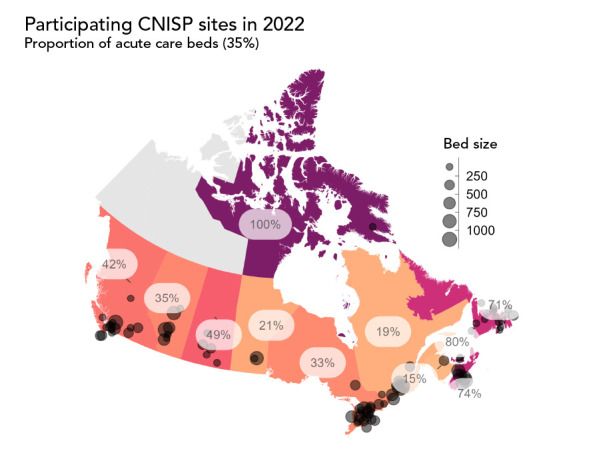
Geographical distribution and characteristics of the Canadian Nosocomial Infection Surveillance Program participating hospitals across Canada^a,b^ Abbreviation: CNISP, Canadian Nosocomial Infection Surveillance Program ^a^ Percentage labels represent the percentage of acute care beds within each province/territory captured by CNISP ^b^ Circles represent CNISP participating hospitals. The size of the circle is proportional to the hospital’s bed capacity

In 1995, CNISP conducted active surveillance in 18 hospitals across seven provinces and reported on only one antibiotic-resistant organism (ARO): methicillin-resistant *Staphylococcus aureus* (MRSA). By 2022, CNISP has expanded to conduct surveillance on 12 different pathogens in 88 hospitals across 10 provinces and 1 territory. **Figure 1** presents the complete list of pathogens CNISP conducts surveillance on, which includes healthcare-associated infections and AROs, along with the year surveillance of each started. CNISP also annually collects and analyzes data from Canadian hospitals on antimicrobial use (AMU), antibiogram, infection prevention and control (IPC) practises, laboratory practises and viral respiratory illness including coronavirus disease 2019 (COVID-19). **Figure 2** presents the geographical distribution and characteristics of hospitals across Canada participating in CNISP surveillance in 2022.

The expansion of CNISP to include rural, northern and community hospitals has improved the generalizability of its hospital-based surveillance data. As of 2022, one-third of CNISP participating hospitals (n=28/88, 32%) are non-teaching hospitals in the community, as defined by the Canadian Institute for Health Information (4). Further, the number of beds across the 88 hospitals participating in CNISP surveillance in 2022 ranged from 3 to 1,087 and 1 of 3 territories are represented. In addition to improvements in CNISP representativeness, the scope of CNISP has expanded. CNISP began collecting data on community-associated (CA) MRSA in 2010 and has since expanded to collect data on CA infections (e.g. CA *Clostridioides difficile* infections; CDI) and AROs (e.g. CA carbapenemase-producing *Enterobacterales*; CPE). Other areas in which CNISP has expanded its scope is with surveillance of outpatient CDI and emerging pathogens such as *Candida auris*.

### Functions

#### Collect and analyze data

CNISP is the only national hospital sentinel system in Canada that actively collects AMR data via standardized methods. Definitions and protocols, which are publicly available online, facilitate this standardized data collection. CNISP analyzes demographic and clinical data abstracted from patient charts by trained IPC professionals, with linked molecular and microbiological data abstracted from centralized laboratory testing conducted by the National Microbiology Laboratory. A major strength of CNISP, relative to other surveillance systems, is its integration of these data. This comprehensive dataset has been essential in the monitoring of emerging AMR pathogens, including, for example, the hyper virulent *C. difficile* NAP1 (rt027) strain type, the emergence of CA-MRSA strain types (CMRSA10/USA300 and CMRSA7/USA400), vancomycin-resistant *Enterococci* (VRE) sequence type 1478 and CPE (5-9).

#### Provide benchmarks

A key function of CNISP is to provide participating hospitals and knowledge users, such as IPC and antimicrobial stewardship professionals, with benchmarks for hospital-acquired infection, ARO and AMU rates. By comparing their own site-specific rates to regional and national rates, participating hospitals can assess their progress in AMR prevention and intervene as needed. To facilitate this for selected surveillance projects, such as AMU, CNISP has developed and automated a site-specific report that presents site-specific rates relative to the rates of comparable hospitals in the CNISP network (de-identified in the site-specific report). In addition, participating hospitals have access to visual analytics for CDI on the Canadian Network for Public Health Intelligence platform, the secure on-line platform where hospitals submit their data. The Canadian Network for Public Health Intelligence visual analytics offers CNISP hospitals the ability to compare their rates of CDI to hospitals similar in size, type (community vs. teaching) or services offered, and to regional, provincial and national rates. Hospitals can also view resistance profiles and molecular characteristics (e.g. ribotypes). Antimicrobial stewardship groups, administrators and IPC staff may further benefit by utilizing these hospital-based surveillance data to guide quality improvement initiatives that tackle AMR, such as reducing AMU or implementing bundled interventions to reduce the risk of infection.

#### Disseminate scientific evidence

Since 1995, in collaboration with the National Microbiology Laboratory and stakeholders from participating hospitals, CNISP has produced over 260 publications, including peer-reviewed articles, reports and conference abstracts. These provide scientific evidence to inform public health action to reduce AMR. CNISP annually publishes reports summarizing trends in healthcare-associated infections and AMR in the *Canada Communicable Disease Report* and on the Government of Canada website. To improve accessibility and uptake of CNISP surveillance data among the public and healthcare professionals outside of the CNISP network, in 2022, CNISP launched an interactive data blog on the Government of Canada website. These data are consistent with those reported in the *Canada Communicable Disease Report* and additionally include data pertaining to AMU in hospitals, demonstrating CNISP’s progress towards achieving integrated AMR/AMU surveillance across Canadian hospitals. This publicly available interface provides timely data syntheses and interactive visualizations to inform strategies to combat AMR sooner than peer-reviewed publications.

#### Guide policy and practice

CNISP surveillance data informs evidence-based policy and guidelines within Canada and internationally. For example, the Manitoba provincial government applies CNISP standardized definitions in their CDI clinical management protocol (10). Further, CNISP hospital-based surveillance informed provincial guidelines for the prevention and control of AROs (11). The CNISP supports the collaborative work plan of the Public Health Agency of Canada as demonstrated by its international partnerships with the Transatlantic Taskforce on Antimicrobial Resistance and the World Health Organization Global Antimicrobial Resistance and Use Surveillance System. CNISP provides antibiogram data to the Global Antimicrobial Resistance and Use Surveillance System for incorporation into their international database and report, which provide insights into the global burden of AMR (12). In addition, CNISP contributes hospital-based AMR data to the Canadian Antimicrobial Resistance Surveillance System annual report, which presents human data from CNISP with data from the animal, environmental and food safety sectors (13).

#### Adapt to public health needs

At the start of the COVID-19 pandemic, CNISP leveraged its existing network of sentinel hospitals across Canada to expand the scope of its viral respiratory illness surveillance to include CA and healthcare-associated COVID-19. CNISP participating hospitals collect COVID-19 patient level data, including demographic, clinical, outcome, AMU and ARO co-infection data. Using these patient level data, CNISP published a peer-reviewed article describing the epidemiology of patients with COVID-19 admitted to CNISP participating hospitals (14). Currently, CNISP is analyzing the impact of COVID-19 on ARO rates calculated from CNISP hospital-based surveillance data to better understand how the burden of AMR in hospitals has changed in Canada. CNISP also demonstrated its adaptability to respond to new and emerging pathogens by way of its initiation of *C. auris* surveillance in 2019. Since then, CNISP has contributed to understanding the prevalence of *C. auris* in Canadian acute-care hospitals and preparedness for *C. auris* in CNISP participating hospitals (15,16).

## Discussion

For more than 20 years, CNISP has been a successful collaboration between the federal government, national organizations and sentinel hospitals across Canada. In the future, CNISP will seek to recruit hospitals from the Northwest Territories and provinces with currently low representation. To further increase participation and improve the representativeness of its hospital-based surveillance data, CNISP has launched a Simplified Dataset (SDS). The SDS uses CNISP standardized definitions and aims to capture data on healthcare-associated infections and AROs from acute-care hospitals outside of the CNISP network. While hospitals participating in CNISP active surveillance submit patient-level data, hospitals participating in the SDS submit aggregate data (annual number of cases, patient days and patient admissions). In combining both data sources, CNISP will be able to report national and regional rates of AMR from a greater number and more representative sample of Canadian hospitals. After successful pilot testing of the SDS for CDI surveillance, CNISP is seeking to recruit additional hospitals outside of the network to participate in the SDS for CDI surveillance.

To further describe the burden of AMR in Canadian hospitals, CNISP will be conducting a point prevalence survey in 2023, which aims to include acute-care hospitals within and outside of the CNISP network. This survey will build upon three-point prevalence surveys conducted in 2002, 2009 and 2017 by CNISP. For Canadian hospitals, these repeated surveys are widely utilized to benchmark hospital-acquired infection, ARO and AMU rates, measure changes in prevalence over time, provide information on AMR control programs and identify new targets for surveillance (17-19). CNISP also seeks to expand its use of whole-genome sequencing to enable a deeper analysis of the evolving molecular epidemiology and transmission of AMR pathogens in Canada. Data from whole-genome sequencing can support IPC and stewardship practises in hospitals, and ultimately enhance public health interventions for AMR and infectious diseases (20).

Because CNISP is a hospital-based surveillance system, its AMR and AMU data are not generalizable to settings such as primary and long-term care. To improve our understanding of AMR in Canada, future surveillance efforts should focus on ascertaining AMR and AMU data from these under-represented settings (3,21). While CNISP captures data on CDI in outpatient settings and CA AROs, such as CA MRSA, CA CPE, CA VRE and CA CDI, there remains an important gap in our understanding of AMR and AMU in community settings (3,21). To help address this, future expansion of CNISP also includes the initiation of AMR surveillance in long-term care. The scope and methodology for long-term care surveillance are currently under development.

### Conclusion

Supported by the federal government, CNISP is a core national program that has monitored AMR in Canadian acute-care hospitals since 1995. Surveillance data from this network of urban and community hospitals across Western, Central, Eastern and Northern Canada is used to provide benchmarks and inform evidence-based action, such as antimicrobial stewardship. Given its achievements in recent years and future directions, CNISP is well positioned to serve as the reference point for hospital-based AMR data in Canada.

